# Prison‐based interventions are key to achieving HCV elimination among people who inject drugs in New South Wales, Australia: A modelling study

**DOI:** 10.1111/liv.15469

**Published:** 2022-11-14

**Authors:** Jack Stone, Aaron G. Lim, Gregory J. Dore, Annick Borquez, Louise Geddes, Richard Gray, Jason Grebely, Bezhad Hajarizadeh, Jenny Iversen, Lisa Maher, Heather Valerio, Natasha K. Martin, Matthew Hickman, Andrew R. Lloyd, Peter Vickerman

**Affiliations:** ^1^ Population Health Sciences, Bristol Medical School University of Bristol Bristol UK; ^2^ The Kirby Institute, UNSW Sydney New South Wales Sydney Australia; ^3^ Division of Infectious Diseases and Global Public Health University of California San Diego California USA; ^4^ NIHR Health Protection Research Unit in Behavioural Science and Evaluation at University of Bristol Bristol UK

**Keywords:** elimination, hepatitis C virus, incarceration, people who inject drugs

## Abstract

**Background & Aims:**

People who inject drugs (PWID) experience high incarceration rates which are associated with increased hepatitis C virus (HCV) transmission risk. We assess the importance of prison‐based interventions for achieving HCV elimination among PWID in New South Wales (NSW), Australia.

**Methods:**

A model of incarceration and HCV transmission among PWID was calibrated in a Bayesian framework to epidemiological and incarceration data from NSW, incorporating elevated HCV acquisition risk among recently released PWID. We projected the contribution of differences in transmission risk during/following incarceration to HCV transmission over 2020–2029. We estimated the past and potential future impact of prison‐based opioid agonist therapy (OAT; ~33% coverage) and HCV treatment (1500 treatments in 2019 with 32.9%–83.3% among PWID) on HCV transmission. We estimated the time until HCV incidence reduces by 80% (WHO elimination target) compared to 2016 levels with or without prison‐based interventions.

**Results:**

Over 2020–2029, incarceration will contribute 23.0% (17.9–30.5) of new HCV infections. If prison‐based interventions had not been implemented since 2010, HCV incidence in 2020 would have been 29.7% (95% credibility interval: 22.4–36.1) higher. If current prison and community HCV treatment rates continue, there is an 98.8% probability that elimination targets will be achieved by 2030, with this decreasing to 10.1% without current prison‐based interventions.

**Conclusions:**

Existing prison‐based interventions in NSW are critical components of strategies to reduce HCV incidence among PWID. Prison‐based interventions are likely to be pivotal for achieving HCV elimination targets among PWID by 2030.

**Lay Summary**

- The added risk associated with incarceration results in it contributing a fifth of all new HCV transmission among people who inject drugs (PWID) in New South Wales (NSW, Australia).
- Existing prison‐based interventions (opioid agonist therapy and HCV treatment) have reduced HCV incidence among PWID by a quarter.
- Prison‐based interventions particularly HCV treatment, are crucial for achieving HCV elimination targets by 2030, with the probability that elimination occurs by 2030 reducing from 99% to 10% if current prison‐based interventions are ceased.

## 1 INTRODUCTION

Over half of people who inject drugs (PWID) globally have been exposed to hepatitis C virus (HCV),^1^ with injecting drug use (IDU) being the leading cause of HCV infection in developed countries.^2^

Modelling studies suggest that disease elimination is achievable through the scale‐up of highly effective direct‐acting antiviral (DAA) therapies for chronic HCV infection.^3^, ^4^ In 2016, this led the World Health Organization (WHO) to set a goal of eliminating HCV as a public health threat by 2030. PWID need to be a high priority for elimination efforts. However, poor coverage of harm reduction measures, restricted access to DAAs, and criminalization of drug use are critical barriers to achieving HCV elimination among PWID.^5^

Evidence suggests that incarceration can be an important driver of HCV transmission among PWID due to high incarceration rates (>50% ever^1^) and heightened risk during incarceration and/or post‐release.^6^, ^7^, ^8^ Our recent systematic review suggests that among community PWID recent incarceration is associated with a 62% increased risk of HCV acquisition,^9^ increasing to 180% in Australia.^9^

Prison‐based interventions can have substantial impact on the overall HCV epidemic among PWID.^7^, ^8^ In NSW prisons, Opioid agonist therapy (OAT) has a high coverage (33%^10^), comparable to the community coverage (40%).^11^ Recognition of the importance of the prison sector for HCV transmission and an opportunity for DAA treatment scale‐up, has also led to specific initiatives to ensure universal access to DAAs for prisoners in Australia. While prisoners only constituted 6% of individuals initiating DAA therapy in 2016, this has risen progressively to 29% in 2019.^12^

We use a model of HCV transmission and incarceration among PWID in NSW to assess the contribution of incarceration to HCV transmission, evaluate the historical impact of prison‐based OAT and HCV treatment, and determine their importance for achieving WHO elimination target of reducing HCV incidence by 80% by 2030.

## 2 METHODS

### 2.1 Model description

**FIGURE 1:**
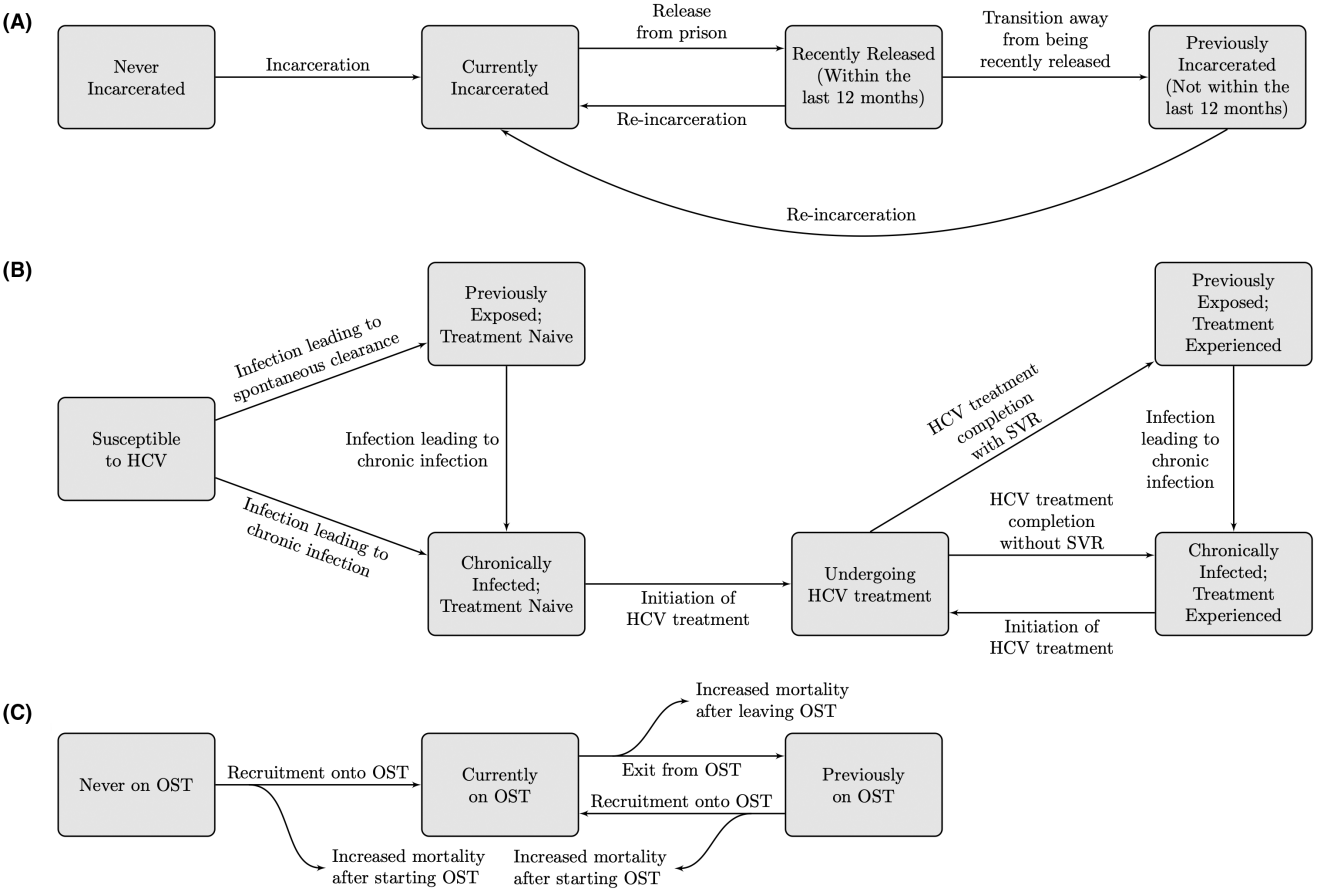
Model schematics of the model components of: (A) incarceration, (B) HCV infection and treatment and (C) transitions on and off OAT. Model component of injecting duration is not shown.

We adapted a published^8^ dynamic, deterministic model of incarceration and HCV transmission among current PWID, stratified by injecting duration, incarceration state, HCV infection state and OAT status (Figure 1).

PWID enter the model through initiating injecting drug use, balanced by PWID leaving through permanent cessation of injecting (varies over time and by injecting duration) or death. PWID on OAT experience reduced mortality.^13^ New PWID are susceptible and not on OAT, with fixed proportions entering each incarceration state. PWID transitions through injecting duration states until experiencing injecting cessation or death.

PWID initiate and leave OAT at constant rates, with excess overdose mortality at these points^14^ and higher recruitment rates if they have been on OAT before. PWID are incarcerated or re‐incarcerated at different rates (to capture greater rates of re‐incarceration than primary incarceration), varying by injecting duration, and are released at a constant rate. PWID experience reduced rates of incarceration while on OAT.^15^ Two‐thirds of PWID are retained on OAT upon release.^16^

PWID transmit HCV in their given setting (prison or community). Transmission occurs at a rate proportional to the chronic prevalence in each setting and the infection rate. The infection rate differs by setting, and is elevated among PWID with <3 years of injecting^17^, ^18^ or who have been recently released from prison,^9^ and is reduced if on OAT.^19^ Most new infections lead to chronic infection.^20^

A time‐varying number of chronically infected PWID are treated annually. Community PWID on OAT are more likely to be treated than those not on OAT.^11^, ^21^ A proportion of treated PWID achieve sustained viral response (SVR). Those failing treatment are eligible for retreatment due to unrestricted access in Australia.

### 2.2 Model parameterization and calibration

Data for parameterizing and calibrating the model came primarily from the ANSPS Survey,^11^ an annual cross‐sectional sero‐behavioural survey conducted among PWID attending needle and syringe programmes (NSP) across Australia since 1995, and the SToP‐C study (2014–2019), which assessed the feasibility of HCV treatment as prevention in the prison setting.^10^ Other data sources were used to validate the model, including four sero‐behavioural cross‐sectional studies of prison entrants (NPEBBVS, 2004, 2007, 2010 and 2013^22^), an observational study of the HCV care cascade among PWID (ETHOS Engage study^21^), and HCV incidence estimates from two longitudinal studies of PWID in the community (HITS‐c, 2008–2012^18^) and prison (HITS‐p, 2005–2014^23^).

**FIGURE 2:**
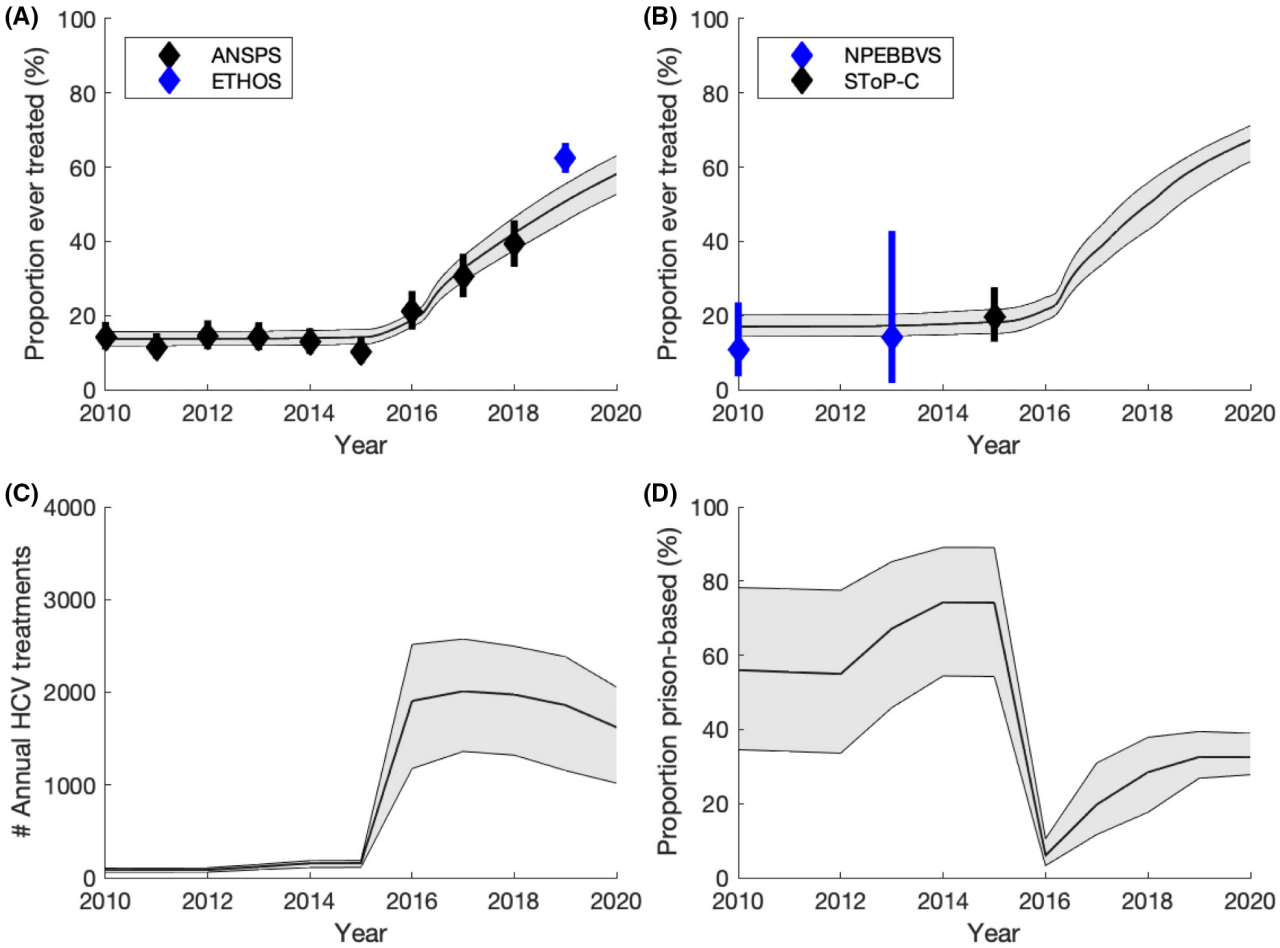
Modelled HCV treatment uptake over time: (A) Proportion of HCV antibody‐positive PWID in the community who have ever been treated; (B) Proportion of HCV antibody‐positive PWID in prison who have ever been treated; (C) Annual number of HCV treatment initiations; (D) Proportion of treatment initiations in prison. All lines show the median model projections, while the shaded area shows the 95% CrI for the baseline projections. Data points with their 95% CI are shown for comparison; data‐points shown in (b) were not used for model calibration.

We assume that the incarceration dynamics, HCV epidemic and demographics of PWID were stable in 2010 based on data from ANSPS. OAT initiation rates were calibrated to give stable coverages of 29%–37% among incarcerated PWID and 40%–52% among community PWID. The rate of HCV treatment among community PWID is assumed constant until 2015, and then calibrated to the increasing proportion of antibody‐positive PWID reporting ever receiving HCV treatment in ANSPS (Figure 2A). The annual number of PWID treated in prison is assumed proportional to the total number of treatments started in NSW prisons (increases from 80 to 1500 per year over 2010–2018), assuming a fixed proportion among current PWID. The model assumes a factor increase in community HCV acquisition risk among PWID recently released from prison (2.78, 95% CI: 2.00–3.85), based on Australian data.^9^ The HCV transmission risk in prison is calibrated to give the difference in HCV antibody prevalence among community PWID with and without a history of incarceration.

The model is calibrated using an approximate Bayesian computation sequential Monte Carlo algorithm (see Appendix S1). The algorithm begins with 1000 parameter sets sampled from prior distributions (Table 1), which are then resampled and perturbed in an iterative manner to better fit the data until the goodness of fit (measured using log‐likelihood) no longer improves (<0.5% relative difference between successive iterations).

**TABLE 1:** Prior distributions for model parameters

Parameter	Prior distribution	Source/Notes
Demographic parameters		
Proportion of PWID who start injecting in prison	Uniform with range 0%–30%	Priors based on % of PWID with <3 years injecting who have never been incarcerated. Calibrated to: % incarcerated PWID that first injected in prison.
Proportion of PWID who start injecting in the community who have never been incarcerated	Uniform with range 70%–100%	
Proportion of PWID who start injecting in prison who are incarcerated for the first time	Uniform with range 0%–100%	
Mortality rate—out of OAT (per 1000 pys)	Normal with mean 8.9 and 95% CI: 8.6–9.2	NSW data^24^
Average cessation rate per year (<2010)		Calibrated to % community PWID in each injecting duration category over time.^25^
<3 years injecting	Uniform with range 0–0.3	
3–15 years injecting	Uniform with range 0–0.1	
15+ years injecting	Uniform with range 0–0.1	
Factor change in cessation rate after 2010 if injecting 15+ years injecting	Uniform with range 0–1	
Incarceration parameters		
Annual incarceration rate among PWID <3 years injecting	Uniform with range 0–1	Calibrated to: % community PWID that have ever been incarcerated by duration of injecting; % incarcerated PWID that have ever been incarcerated before, by duration of injecting.
Annual incarceration rate among PWID > = 3 years injecting	Uniform with range 0–1	
Annual re‐incarceration rate among PWID <3 years injecting	Uniform with range 0–1	
Annual re‐incarceration rate among PWID ≥3 years injecting	Uniform with range 0–3.0	
Average duration of each incarceration (months)	Uniform with range 4–6	Mean length of adult incarceration episodes among opioid‐dependent people who have ever been registered for OAT is 5.1 months. Assume average duration for PWID is similar with additional uncertainty.^26^
Relative risk of (re‐)incarceration rates if on OAT	Lognormal with mean 0.79 and 95% CI: 0.70–0.89	^15^
HCV transmission parameters		
Baseline HCV Transmission rate	Uniform with range 0–1.0	Calibrated to HCV antibody prevalence among community PWID by incarceration history (ever or never incarcerated).
Relative increase in HCV transmission risk if currently incarcerated PWID	Uniform with range 0–5	
Relative increase in HCV transmission risk among recently released PWID (12 months since release)	Lognormal with mean 2.78 and 95% CI 2.00–3.85	Australian studies from systematic review (ANSPS retrospective cohort and ANSPS 2012).^9^
Relative increase in HCV transmission risk if <3 years injecting	Lognormal with mean 1.72 and 95% CI 0.94–2.51	17, 18
Relative reduction in HCV transmission if on OAT	Lognormal with mean 0.50 and 95% CI: 0.40–0.63	Assume same effect in prison as the community.^19^
Proportion of new infections that spontaneously clear	Lognormal with mean 0.26 and 95% CI: 0.22–0.29	^20^
HCV treatment parameters		
Annual rate of treating HCV‐positive PWID in community		Calibrated to % of Ab+ve PWID in the community who have ever been treated over time.
2010–2012	Uniform with range 0–0.05	
2013	Uniform with range 0–0.05	
2015	Uniform with range 0–0.05	
2018	Uniform with range 0–0.2	
Relative increase in rate of initiating HCV treatment if on OAT	Uniform with range 1–50	Calibrated to OR of ever OAT on ever receiving HCV treatment (2.15, 95% CI: 1.36–3.39).^11^
Number of HCV treatments in prison per year		
<2012	80	Assume all treatments ever are among people with a history of injecting drug use and these are given randomly between those with recent or ever injecting drug use (see proportion in next row).^27^
2012–2014	Linear increase from 80 and 200	
2014–2016	200	
2016	700	
2017	1000	
2018–2020	1500	
Proportion of HCV treatments in prison among current PWID	Range: 32.9–83.3%	Range based on the proportion of prisoners with a history of IDU who recently injected drugs before their incarceration. Range based on estimates from drug use in prison surveys^28^ and prisoner health surveys^29^ including the NSW 2015 Network Patient Health Survey.^30^
Proportion of individuals that achieve sustained viral response		
Before March 2016	Normal with mean 52.6% and 95% CI:44.2–61.0.	Adjusted for genotype distribution.^31^, ^32^
After March 2016	Normal with mean 91.65% and 95% CI: 87.9–95.4.	ITT SVR among recent IDU, with or without OAT.^33^
Average duration of HCV treatment		
Before March 2016	36 weeks	Based on genotype distribution^32^; with 24 weeks for G2/3 and 48 weeks for G1.
After March 2016	12 weeks	
OAT parameters		
Rate of enrolling onto OAT in community if never been on OAT	Uniform with range 0–1	Calibrated to % community PWID currently on OAT over time; % community PWID ever in OAT over time
Relative increase in OAT recruitment rate if have previously been on OAT compared to never been on OAT	Uniform with range 1–100	
Rate of enrolling onto OAT in prison if never been on OAT	Uniform with range 0–1	Calibrated to % incarcerated PWID currently on OST
Average duration on OAT (months)	Uniform with range 6.5–8.75	NSW data^34^
Relative risk of all‐cause mortality if on OAT	Lognormal with mean 0.33 and 95% CI: 0.28–0.39	^13^
Relative risk of all‐cause mortality		
In the first 4 weeks after starting treatment compared to on OAT	Lognormal with mean 1.97 and 95% CI: 0.94–4.10	^14^
In the first 4 weeks after leaving treatment compared to off OAT	Lognormal with mean 2.38 and 95% CI: 1.51–3.74	^14^

The model is calibrated to the: proportion of community PWID who have ever been incarcerated by duration of injecting; proportion of incarcerated PWID who are incarcerated for first time by duration of injecting; proportion of incarcerated PWID who first injected in prison; HCV antibody prevalence among community PWID by incarceration history (never or ever); proportion of community PWID in each duration of injecting categories; proportion of antibody‐positive community PWID who have ever been treated; increased odds of ever receiving HCV treatment if ever on OAT compared to never on OAT; OAT coverage among PWID in the community and in prisons. To calibrate the incarceration and re‐incarceration rates in the full model, an incarceration sub‐model was used alongside the full model, which simulated a cohort of PWID stratified by finer injecting duration categories (3‐year intervals) than the full model (<3, 3–15 and 15+ years). Calibration data and the model fits are in the supplementary materials.

The model was validated against data on the number of individuals with recent drug dependence treated for HCV over 2016–2018,^35^ HCV incidence among incarcerated PWID,^23^ HCV antibody and viraemic prevalence among incarcerated PWID (SToP‐C and NPEBBVS^22^) and HCV viraemic prevalence in the community.^11^, ^21^

### 2.3 Model analyses

Using the calibrated model, we projected the contribution (population attributable fraction, ‘PAF’) of incarceration to HCV transmission among PWID over 10 years from 2020 to 2029, calculated as the relative decrease in the number of new HCV infections over that period if the prison HCV transmission risk was set to the same as the community with no excess risk among recently released PWID. We assumed the same effect of prison‐based interventions.

To calculate the historical (2010–2019) impact of prison‐based OAT and HCV treatment, we compared the ‘status quo’ HCV epidemic projections with alternative counterfactual model scenarios that had no prison‐based OAT and/or HCV treatment from 2010. We estimated the number of infections averted and reductions in chronic HCV prevalence and HCV incidence achieved by the interventions included in the status quo model.

Assuming existing coverage levels of OAT and rates of initiating treatment continue to 2030, the status quo model was used to project the reductions in chronic HCV incidence that could occur by 2030 compared to 2016 levels (reference for WHO elimination targets). Alternative scenarios considered the impact of no prison‐based OAT or HCV treatment from 2020. We then projected the impact of scaling‐up or introducing new prison‐based interventions:

#### 2.3.1 Scenario 1

Scale‐up prison‐based HCV treatment rates by 44% in 2020 as achieved by the SToP‐C intervention study in NSW prisons.^10^

#### 2.3.2 Scenario 2

Introduce NSP into prison at 50% or 100% coverage, assuming NSP reduces HCV transmission risk by 56% (95% CI 20%–76%).^19^ This is modelled by reducing the force of infection in prison based on the coverage and effectiveness of NSP (see Appendix S1).

For each scenario, we projected the date by which an 80% reduction in HCV incidence (compared to 2016) could be reached among all PWID and the probability (estimated by proportion of runs) of achieving this target by 2030. In sensitivity analyses, we also considered how the contribution of prison‐based interventions to HCV elimination would differ if community treatment rates reduced by 25% from 2020.

#### 2.3.3 Uncertainty analysis

A linear regression analysis of covariance was undertaken to determine which parameter uncertainties contribute most to uncertainty in the impact of prison‐based interventions to reducing HCV incidence over 2020–2030. The proportion of each model outcome's sum‐of‐squares contributed by each parameter is used to estimate the importance of individual parameters to the overall uncertainty.

## 3 RESULTS

### 3.1 Status quo model projections

**FIGURE 3:**
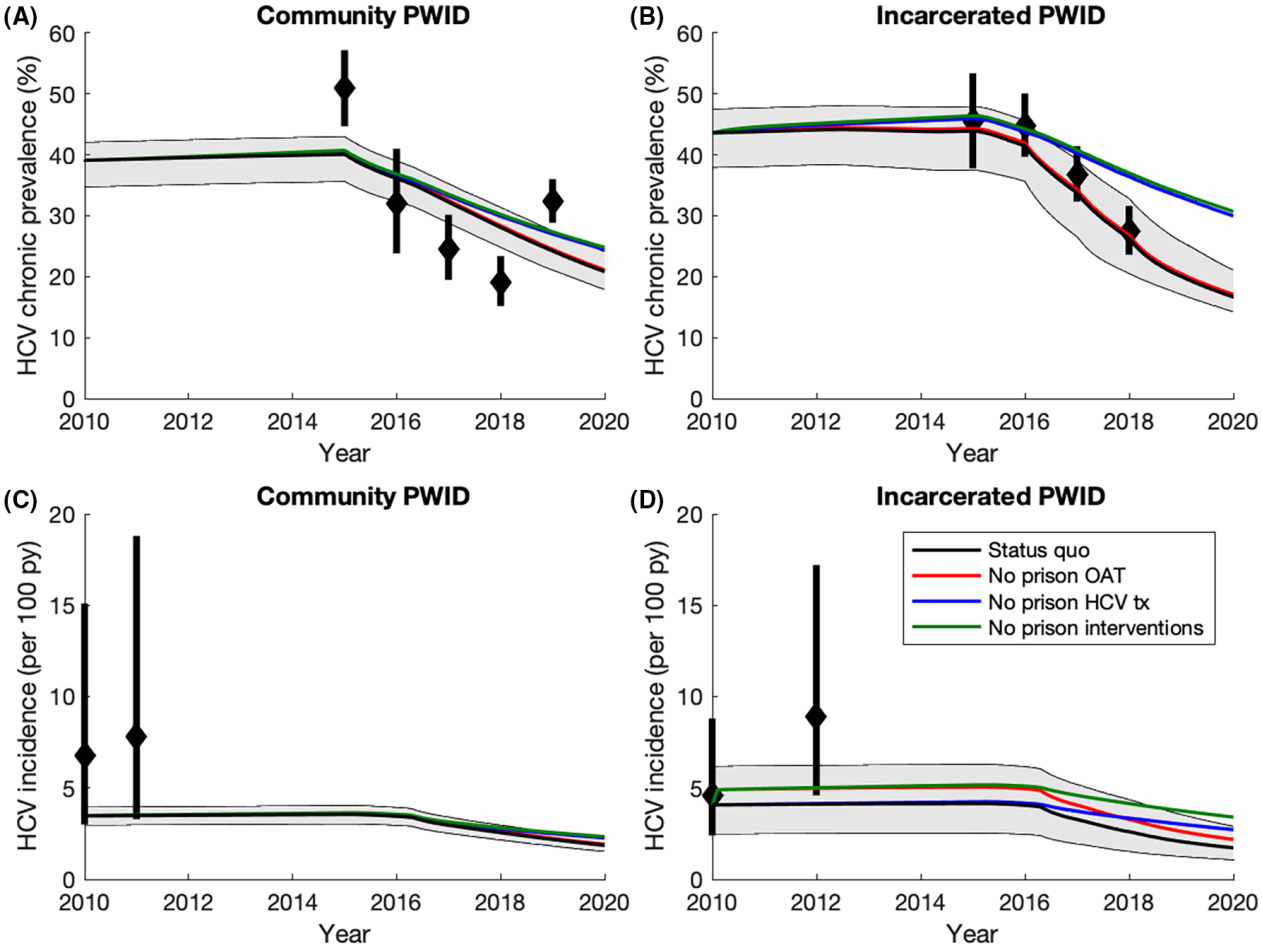
Historical impact of prison‐based interventions on HCV chronic prevalence and incidence among PWID over time: (A) HCV chronic prevalence among community PWID; (B) HCV chronic prevalence among incarcerated PWID; (C) HCV incidence among community PWID; (D) HCV incidence among incarcerated PWID. All lines show the median model projections whilst the shaded area shows the 95% CrI for the baseline projections. Data points with their 95% CI are shown for comparison and were not used for model calibration.

The calibrated model agrees well with the available data (Figure 3 and Appendix S1). Model projections on average fall within 74% of the 95% CI for the four HCV incidence validation points (not fit to) and project that 6328 (95% CrI: 4176‐8109) PWID were treated for HCV between 2016 and 2018 (Figure 2), similar to what was suggested by data (7560^35^).

The model projects an HCV chronic prevalence of 20.5% (17.6–23.9) among PWID in 2020, and an HCV incidence of 1.9 per 100py (1.5–2.2). In 2020, 7.1% (6.2–8.1) of PWID are currently incarcerated and 52.8% (48.7–57.0) have ever been incarcerated. Projections suggest that currently incarcerated PWID have 0.6–1.9 times the transmission risk of PWID in the community (not recently released), while those recently released (in last year) have 2.6–3.8 times the transmission risk. Removing these differences in transmission risk could avert 23.0% (17.9–30.5) of infections over 2020–2029.

### 3.2 Historical impact of prison‐based interventions

In 2020, the model projects OAT coverages of 34.3% (28.2–39.0) and 46.1% (44.2–48.0) in prison and the community respectively. Over 2010–2019, we estimate 10 407 (6810‐13 297) PWID were treated for HCV, with 25.7% (17.8–34.3) initiated in prison (Figure 2). Although prison‐based treatments increase over time, the proportion decreases over 2015–2019 (from 65.7% to 23.5%) due to the scale‐up in community treatment.

In 2019, prison‐based OAT and HCV treatment averted 15.7% (12.2–19.1) of new HCV infections. If these interventions had not occurred since 2010, HCV chronic prevalence would be 22.8% (16.5–27.7) higher in 2020 and incidence would be 29.7% (22.4–36.1) higher (Figure 3). Projections suggest prison‐based HCV treatments have had 4.7 (2.6–6.6) times greater impact on reducing HCV incidence than prison‐based OAT.

### 3.3 Role of prison‐based interventions in HCV elimination

**FIGURE 4:**
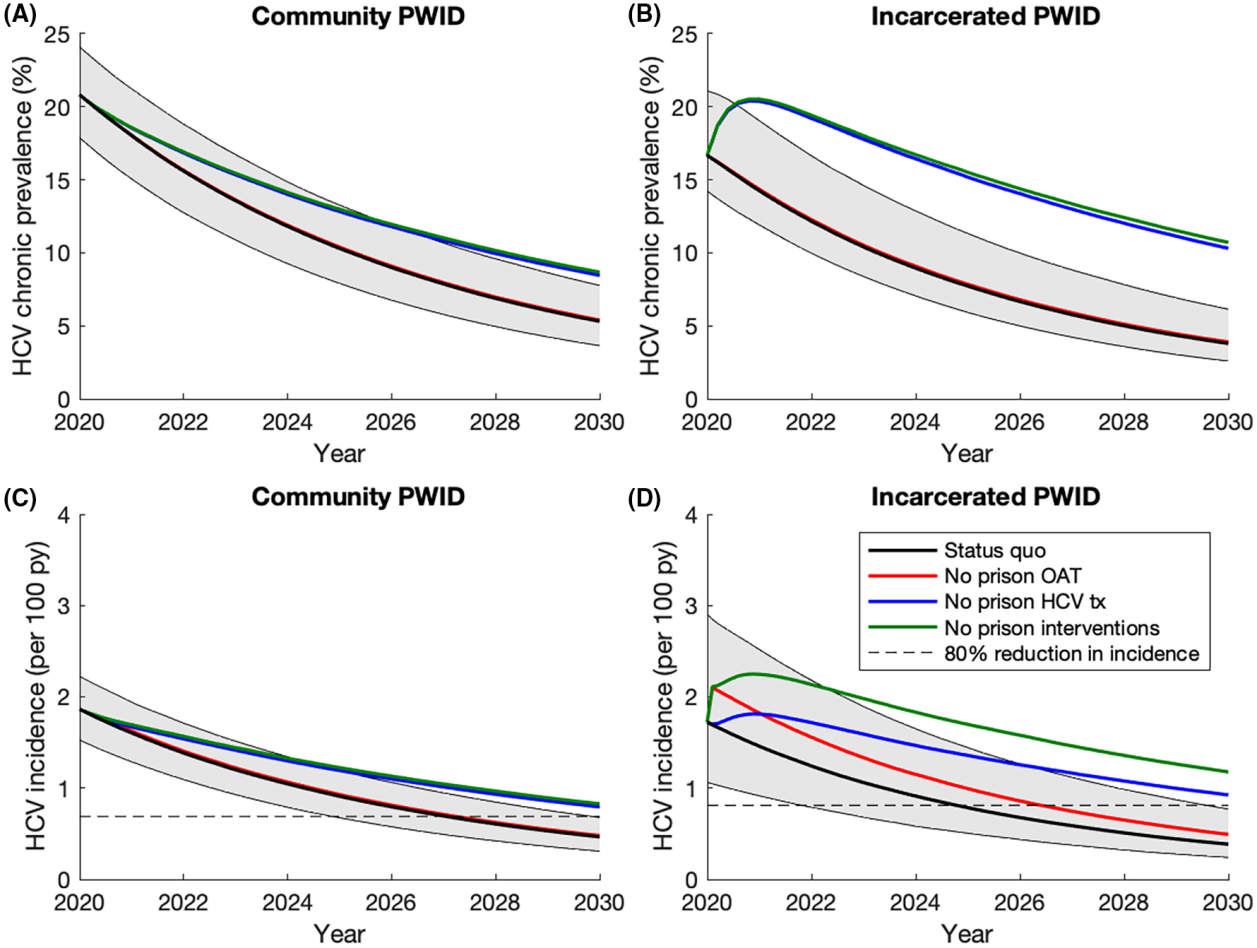
Ongoing impact of prison‐based interventions on HCV chronic prevalence and incidence among PWID over time: (A) HCV chronic prevalence among community PWID; (B) HCV chronic prevalence among incarcerated PWID; (C) HCV incidence among community PWID; (D) HCV incidence prevalence among incarcerated PWID. All lines show the median model projections whilst the shaded area shows the 95% CrI for the status quo projections. Data points with their 95% CI are shown for comparison. Dashed black lines show the level of incidence required to achieve an 80% reduction compared to the median incidence in 2016.

**FIGURE 5:**
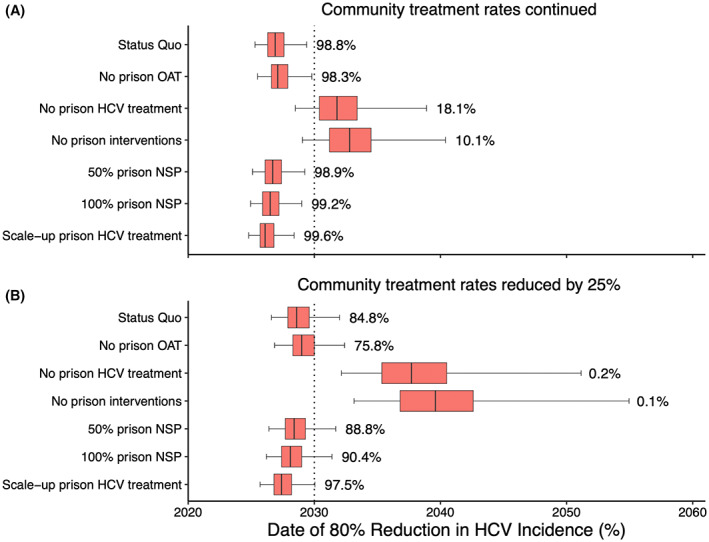
Date of achieving an 80% reduction in overall HCV incidence among PWID compared to 2016 if (A) community treatment rates continue or (B) community treatment rates reduce by 25%. The box plots signify the uncertainty (middle line is median, limits of boxes are the 25% and 75% percentiles, and whiskers are 2.5% and 97.5% percentile range) in the time estimates for achieving elimination due to uncertainty in the model parameters. Labels represent the probability of achieving elimination before 2030.

The *status quo* model projects (Figure 4 and Figure 5) that an 80% reduction in HCV incidence (compared to 2016 levels) will likely be achieved in 2027 (median value; 95% CrI: 2025–2029) if community and prison‐based interventions continue at their current levels, with an 98.8% probability of HCV elimination by 2030. Without prison‐based interventions, the probability of achieving elimination by 2030 reduces to 10.1% and elimination will now occur in 2033 (2029–2040), with removal of prison‐based HCV treatment having the biggest effect (Figure 5). Scaling‐up prison‐based HCV treatment or introducing NSP in prisons from 2020 is unlikely to have much additional effect on achieving elimination if current high levels of community HCV treatment continue. However, if community treatment rates decrease by 25% in 2020, scaling‐up prison‐based HCV treatment or introducing NSP in prisons at 100% coverage from 2020 could increase the probability of elimination from 84.8% to 97.5% or 90.4% respectively.

### 3.4 Uncertainty analysis

Analyses of covariance (Table S1) indicate that uncertainty in community HCV treatment rate from 2015 onwards contributed most (56.2%) to the variability in the contribution of prison‐based interventions to reducing HCV incidence over 2020–2030.

## 4 DISCUSSION

Prison exposure is common among PWID. Our modelling suggests 8% of PWID in NSW were incarcerated in 2020, approximately 50 times greater than the overall national average, with incarceration contributing one‐fifth of ongoing HCV transmission among PWID in this setting. Prison‐based OAT and HCV treatment have mitigated some of this increase in HCV transmission risk associated with incarceration, averting 16% of infections in 2019 and preventing the HCV incidence among PWID from being 30% higher in 2020. Over 2020–2030, prison‐based interventions, particularly HCV treatment, will be pivotal for achieving HCV elimination among PWID, increasing the probability of achieving elimination by 2030 from 10% to 99%.

### 4.1 Strengths and limitations

This study presents the first impact analysis of an ongoing HCV elimination initiative among PWID in the community and prison. Main strengths include the calibration of our model to detailed data in a Bayesian framework and its cross‐validation adding extra plausibility to our projections. Although there are data uncertainties, for example in the duration of injecting or PWID population size, our calibration method captures this uncertainty and propagates it into model projections. Our study also has limitations.

Firstly, our status quo projections assumed that community HCV treatment rates would continue at current rates. The high treatment rates in NSW and across Australia achieved immediately after the introduction of universal access to DAAs included many patients who were waiting for DAAs to become accessible, and easy‐to‐reach patients commencing DAA therapy. This ‘warehouse effect’ is demonstrated by the subsequent decline in HCV treatment numbers in NSW over 2016–2018.^12^ It is unlikely that levels of treatment in 2018 will be sustained, with previous modelling suggesting that improvements in HCV testing rates are needed to maintain the levels of HCV treatment needed to achieve HCV elimination. Indeed, recently reported treatment numbers for 2019 indicate that 11 580 individuals initiated DAA treatments nationally (down from 16 490 in 2018). This largely reflects falling treatment numbers initiated by specialist physicians (in gastroenterology and infectious diseases), which are not being offset by increases in treatments being initiated in primary care settings such as general practices and drug and alcohol clinics. It is likely that the COVID‐19 pandemic will have further impacted on the 2020 treatment rates. To account for these possible decreases, we considered scenarios in which community treatment rates are reduced by 25% from 2020, with these sensitivity analyses showing an increased important for existing and new prison‐based interventions for achieving HCV elimination.

Secondly, we did not model HCV testing and diagnosis; instead we assumed that all chronically infected PWID could be treated with greater treatment rates. We were therefore unable to consider the contribution of prison‐based HCV screening. While most people diagnosed with HCV in prison will begin treatment in prison, this may also lead to increased HCV treatment in the community because the time in custody is typically short (weeks‐months). We therefore may have underestimated the importance of prison‐based interventions for achieving HCV elimination.

Thirdly, our findings may not be generalizable to other settings—either in Australia or internationally, as our model was parameterized and calibrated using detailed epidemiological data from NSW. However, given that NSW has high coverage of harm reduction interventions and high levels of ongoing HCV treatment in the community, it is likely that prison‐based interventions will also be important for achieving HCV elimination among PWID in many other global settings, particularly those with similarly high levels of incarceration or in settings with longer prison sentences.

Finally, although there is uncertainty around the effectiveness of prison‐based OAT, with the HITS‐p study in NSW finding no protective effect on HCV incidence,^23^ the authors noted that OAT likely serves as a surrogate marker for high‐risk behaviours, while the timeliness of commencement on OAT and dosage provided in prison may have been sub‐optimal. Furthermore, the analysis included all cohort participants, including those for whom OAT may not be indicated (e.g. non‐opioid injectors), which may underestimate the protective effect of OAT. International studies have shown that prison‐based OAT is associated with reduced heroin use, injecting and syringe‐sharing,^36^ similar to what is found in community studies. We therefore assumed that prison‐based OAT would be as effective as community OAT^19^; however, if this is not the case, we may have overestimated the impact of prison‐based OAT.

### 4.2 Comparisons with existing studies

Our analysis is consistent with previous modelling that showed incarceration to be an important contributor to HCV transmission in Scotland and Kentucky.^7^, ^8^ However, our estimates are lower than previous illustrative modelling for Australia, which found a PAF of incarceration of 49%,^6^ although that analysis assumed a much higher HCV incidence within prisons and was not calibrated to incarceration or epidemiological data from Australia.

Our results are also consistent with findings from previous modelling studies that show prison‐based prevention and treatment interventions can reduce HCV transmission in the community.^7^, ^8^ However, ours is the first study to directly quantify the importance of ongoing prison‐based interventions for achieving HCV elimination among all PWID. Previous modelling of HCV transmission in NSW prisons found that HCV elimination targets for incidence in the prison setting could be achieved by 2030 via scaling‐up HCV treatments in the community, but that a combination approach of scaled‐up in‐prison HCV treatment and improved harm reduction (OAT and/or NSP) would be required to substantially reduce incidence in prisons by 2030 if community HCV treatment rates reduced.^27^ This study was limited by not mechanistically modelling the interface between community and prison and so was unable to fully capture the effects of interventions in either setting. Other modelling at the national level suggests that existing HCV treatment rates are probably sufficient for Australia achieving HCV elimination targets for incidence,^3^, ^37^ but that testing may need scaling‐up to maintain these treatment rates.^38^ These studies only modelled community PWID and so did not consider the effect of differences in transmission risk or intervention uptake between prisons and the community. Our study therefore extends previous analyses by dynamically modelling HCV transmission in prisons and the community, allowing us to demonstrate the importance of prison‐based interventions for reaching the HCV elimination targets for reducing incidence.

### 4.3 Implications

Australia has committed to eliminating HCV as a public health threat, with the ongoing universal access DAA program achieving high levels of treatment uptake. Our modelling demonstrates that NSW is on track to achieve HCV elimination targets for incidence among PWID by 2030 if current high treatment rates in both the community and prisons continue. Unfortunately, in most other countries prison‐based OAT is unavailable or exists with very low coverage^39^ and most national HCV elimination plans do not encompass interventions for prisoners.^40^ Our modelling demonstrates that even in a setting with high levels of community‐based OAT, NSP and DAA treatment, prison‐based interventions are still important for ensuring HCV elimination targets are achieved. The 2015 United Nations Standard Minimum Rules for the Treatment of Prisoners, known as ‘Nelson Mandela Rules’, directs prison authorities to provide the same standards of healthcare as exists in the community. Thus, focusing HCV elimination efforts solely in the community may not only result in failure but, paradoxically, would breach the human rights of prisoners—one of the populations most affected by the virus.

## CONFLICT OF INTEREST

JG reports grants and personal fees from Abbvie, Gilead Sciences, Merck and Cepheid and grants from Hologic and Indivior, outside the submitted work. MH reports speaker honoraria and travel expenses in last 3 years outside submitted work from MSD and Gilead. PV reports an unrestricted research grant off Gilead Sciences unrelated to this work. AL reports investigator‐initiated research grant support from Abbvie and Gilead Sciences for projects outside the submitted work.

## Supporting information


Appendix S1

## Data Availability

Model code will be made available following publication. The code will be shared with researchers who provide a methodologically sound proposal approved by JS and PV. Proposals should be directed to jack.stone@bristol.ac.uk and peter.vickerman@bristol.ac.uk; requesters will need to sign a data access agreement.
