# Supplementary material for: Prison‐based interventions are key to achieving HCV elimination among people who inject drugs in New South Wales, Australia: A modelling study
Source: Liver Int. 2022 Nov 14;43(3):569–79. doi: 10.1111/liv.15469 (PMC10308445; doi:10.1111/liv.15469)
Supplement: Supplementary file 1 — Appendix S1 [file LIV-43-569-s001.docx]

**Supplementary materials to: Prison-based interventions are key to achieving HCV elimination among people who inject drugs in New South Wales, Australia: a modelling study**

Jack Stone, Aaron G. Lim, Greg J. Dore, Annick Borquez, Louise Geddes, Richard Gray, Jason Grebely, Bezhad Hajarizadeh, Jenny Iversen, Lisa Maher, Heather Valerio, Natasha K. Martin, Matthew Hickman, Andrew R Lloyd, Peter Vickerman

Table of Contents

[Model Equations 2](#_Toc103349208)

[Model Calibration 7](#_Toc103349209)

[Modelling the Introduction of Prison-Based NSP 14](#_Toc103349210)

[Analysis of Covariance 14](#_Toc103349211)

# Model Equations

$X_{i,j}^{m,n}$ is the number of PWID in the model, where

- superscript $m$ denotes incarceration status ($m$ = 1, never incarcerated; $m$ = 2, currently incarcerated; $m$ = 3, recently released from prison (in the last 12 months);$m$ = 4, previously incarcerated but not in the last 12 months)
- superscript $n$ denotes OAT status ($n$ = 1, never on OAT; $n$ = 2, currently on OAT; $n$ = 3, previously on OAT)
- subscript $i$ denotes HCV status ($i$ = 1, Susceptible to HCV; $i$ = 2, Previously exposed – HCV treatment naive; $i$ = 3, chronically infected – treatment naïve; $i$ = 4, HCV treatment; $i$ = 5, Previously exposed – HCV treatment experienced; $i$ = 6, Chronically infected – HCV treatment experienced)
- subscript $j$ denotes injecting duration ($j$ = 1, injecting $<$3 years; $j$ = 2, injecting 3-15 years; $j$ = 3, injecting $>$15 years).

The ordinary differential equations can be written as

$$\frac{dX_{i,j}^{m,n}}{dt}=I_{i,j}^{m,n}+E_{i,j}^{m,n}+A_{i,j}^{m,n}+O_{i,j}^{m,n}+R_{i,j}^{m,n}+H_{i,j}^{m,n}$$

The terms of these differential equations represent different aspects of the model and are described below.

**Inflow of PWID**

$I_{i,j}^{m,n}$ denotes the inflow of new PWID term in the equations and is given by:

$I_{i,j}^{m,n}=\rho^{m}\theta$ if *i=j=n=1*

$I_{i,j}^{m,n}=0$ otherwise

Where,

- $\rho^{m}$ denotes the proportion of new PWID that enter into each incarceration group
- $\sum_{m} \rho^{m}=1$
- $\theta$ denotes the number of new PWID entering the model at time 𝑡

**Injecting cessation and mortality**

$E_{i,j}^{m,n}$ denotes exits from the model through injecting cessation and mortality (N.B. mortality due to elevated risk during the first 4 weeks on or off OAT is included in the transitions on and off OAT subsection), and is given by

$E_{i,j}^{m,n}=-(\mu_{j}+\mu)X_{i,j}^{m,n}$ if $n=1,3$

$E_{i,j}^{m,n}=-(\mu_{j}+\psi\mu)X_{i,j}^{m,n}$ if $n=2$

Where,

- $\mu$ is the rate of mortality if not on OAT
- $\mu_{j}$ is the rate of ceasing injecting for each duration of injecting category $j$
- $\psi$ is the relative risk of mortality if on OAT

**Transitions between injecting durations**

$A_{i,j}^{m,n}$ denotes transitions between injecting durations and is given by

$A_{i,1}^{m,n}=-a_{1}X_{i,1}^{m,n}$

$A_{i,2}^{m,n}=a_{1}X_{i,1}^{m,n}-a_{2}X_{i,2}^{m,n}$

$A_{i,3}^{m,n}=a_{2}X_{i,2}^{m,n}$

Where,

- $a_{j}$ is the rate of transitioning from injecting duration $j$ to $j+1$
- $a_{1}=\frac{1}{3}$ , is the rate of transitioning from <3 years to 3-15 injecting duration
- $a_{2}=\frac{1}{12}$, is the rate of transitioning from 3-15 years to >15 injecting duration

**Transitions on and off OAT**

$O_{i,j}^{m,n}$ denotes transitions between on and off OAT and excess mortality in the first 4 weeks on/off OAT and is given by

$O_{i,j}^{m,1}={-\kappa^{m}X}_{i,j}^{m,1}$

$O_{i,j}^{m,2}={{\left( 1-\mu_{start} \right)\kappa}^{m}(X}_{i,j}^{m,1}+rX_{i,j}^{m,3})-\epsilon X_{i,j}^{m,2}$

$O_{i,j}^{m,3}={-{r\kappa}^{m}X}_{i,j}^{m,3}+\left( 1-\mu_{exit} \right)\epsilon X_{i,j}^{m,2}$

Where,

- $\kappa_{m}$ is the rate of OAT enrolment for OAT naïve PWID in incarceration group $m$with $\kappa_{1}=\kappa_{3}=\kappa_{4}$.
- $r$ is the factor increase in OAT enrolment for OAT experienced PWID compared to OAT naïve PWID.
- $\epsilon$ is the rate of leaving OAT
- $\mu_{start}$ is the proportion of PWID that die due to increased mortality in the first four weeks after initiating OAT and is given by $\mu_{start}=\frac{4}{52}\left( \psi_{s}-1 \right)\psi\mu$
- $\psi_{s}$ is the relative risk of mortality in the first 4 weeks of OAT compared to the rest of time on OAT.
- $\mu_{exit}$ is the proportion of PWID that die due to increased mortality in the first four weeks after exiting OAT and is given by $\mu_{exit}=\frac{4}{52}\left( \psi_{e}-1 \right)\mu$
- $\psi_{e}$ is the relative risk of mortality in the first 4 weeks after leaving OAT compared to the rest of time off OAT.

**Transitions between incarceration states**

$R_{i,j}^{m,n}$ denotes transitions between incarceration states and is given by

$R_{i,j}^{1,n}=-\gamma_{j}X_{i,j}^{1,n}$ if n=1,3

$R_{i,j}^{1,n}=-f\gamma_{j}X_{i,j}^{1,n}$ if n=2

$R_{i,j}^{2,n}=\gamma_{j}X_{i,j}^{1,n}+\delta_{j}(X_{i,j}^{3,n}+X_{i,j}^{4,n})-\tau X_{i,j}^{2,n}$ if n=1,3

$R_{i,j}^{2,n}={f\gamma}_{j}X_{i,j}^{1,n}+{f\delta}_{j}(X_{i,j}^{3,n}+X_{i,j}^{4,n})-\tau X_{i,j}^{2,n}$ if n=2

$R_{i,j}^{3,1}=-\left( \delta_{j}+q \right)X_{i,j}^{3,1}+{\tau X}_{i,j}^{2,1}$

$R_{i,j}^{3,2}=-\left( {f\delta}_{j}+q \right)X_{i,j}^{3,2}+{\chi\tau X}_{i,j}^{2,2}$

$R_{i,j}^{3,3}=-\left( \delta_{j}+q \right)X_{i,j}^{3,3}+{\tau[X}_{i,j}^{2,3}+(1-\chi)(1-\mu_{exit})X_{i,j}^{2,2}]$

$R_{i,j}^{4,n}=-\delta_{j}X_{i,j}^{4,n}+qX_{i,j}^{3,n}$ if n=1,3

$R_{i,j}^{4,n}=-f\delta_{j}X_{i,j}^{4,n}+qX_{i,j}^{3,n}$ if n=2

Where,

- $\gamma_{j}$ is the rate of incarceration for PWID not on OAT with injecting duration $j$
- $f$ is the relative risk of incarceration/reincarceration if on OAT compared to off OAT
- $\delta_{j}$ is the rate of reincarceration for PWID not on OAT with injecting duration $j$
- $\tau$ is the rate of release from prison
- $\chi$ is the proportion of PWID that are retained on OAT when released from prison
- $q$=1 and is the rate of transitioning from being recently released ($m=3$) to non-recently released ($m=4$).

**HCV Transmission and Treatment**

$H_{i,j}^{m,n}$ denotes transitions between HCV infections and treatment states and is given by

$H_{1,j}^{m,n}=-\Lambda_{j}^{m,n}X_{1,j}^{m,n}$

$H_{2,j}^{m,n}=-\left( 1-\alpha\right)\Lambda_{j}^{m,n}X_{2,j}^{m,n}+\alpha\Lambda_{j}^{m,n}X_{1,j}^{m,n}$

$H_{3,j}^{m,n}=\left( 1-\alpha\right)\Lambda_{j}^{m,n}{(X}_{1,j}^{m,n}+X_{2,j}^{m,n})-\phi^{m,n}X_{3,j}^{m,n}$

$H_{4,j}^{m,n}=\phi^{m,n}{(X}_{3,j}^{m,n}+X_{6,j}^{m,n})-\omega X_{4,j}^{m,n}$

$H_{5,j}^{m,n}=\pi\omega X_{4,j}^{m,n}-\left( 1-\alpha\right)\Lambda_{j}^{m,n}X_{5,j}^{m,n}$

$H_{6,j}^{m,n}=\left( 1-\pi\right)\omega X_{4,j}^{m,n}+\left( 1-\alpha\right)\Lambda_{j}^{m,n}X_{5,j}^{m,n}-\phi^{m,n}X_{6,j}^{m,n}$

Where,

- $\Lambda_{j}^{m,n}$ is the force of infection (see below) for PWID with injection duration $j$, incarceration status $m$ and OAT status $n$
- $\alpha$ is the proportion of infections that spontaneously clear
- $\phi^{m,n}$ is the HCV treatment initiation rate for PWID with incarceration status $m$ and OAT status $n$
- $\pi$ is the proportion of treatments that result in SVR
- $\frac{1}{\omega}$ is the average duration of HCV treatment

**Force of Infection**

$\Lambda_{j}^{m,n}$ is the force of infection (see below) for PWID with injection duration $j$, incarceration status $m$ and OAT status $n$, and is given by

$\Lambda_{j}^{m,n}=\eta_{j}^{m,n}\beta_{c}$ if m=1,3,4

$\Lambda_{j}^{m,n}=\eta_{j}^{m,n}\beta_{p}$ if m=2

Where,

$$\beta_{c}=\lambda_{c}\frac{\sum_{n} \sum_{j} \sum_{m=1,3,4} \eta_{j}^{m,n}(X_{3,j}^{m,n}+X_{6,j}^{m,n})}{\sum_{i} \sum_{n} \sum_{j} \sum_{m=1,3,4} \eta_{j}^{m,n}X_{i,j}^{m,n}}$$

$$\beta_{p}=\lambda_{p}\frac{\sum_{n} \sum_{j} \eta_{j}^{2,n}(X_{3,j}^{2,n}+X_{6,j}^{2,n})}{\sum_{i} \sum_{n} \sum_{j} \eta_{j}^{2,n}X_{i,j}^{2,n}}$$

$$\eta_{j}^{m,n}=\upsilon_{j}\zeta_{m}\Omega_{n}$$

And

- $\lambda_{c}$ is the HCV transmission rate in the community for PWID never incarcerated and off OAT.
- $\lambda_{p}$ is the HCV transmission rate in prison for PWID off OAT.
- $\upsilon_{1}$ is the relative transmission risk for PWID with injecting duration <3 years ($j=1)$ compared to PWID with injecting duration 3-15 years ($j=2)$ or >15 years ($j=3)$.
- $\upsilon_{2}=\upsilon_{3}=1$
- $\zeta_{3}$ is the relative transmission risk for PWID who have been recently released from prison ($m=3$) compared to other incarceration states.
- $\zeta_{1}=\zeta_{2}=\zeta_{4}=1$
- $\Omega_{2}$ is the relative transmission risk for PWID on OAT ($n=2)$ compared to those not on OAT.
- $\Omega_{1}=\Omega_{3}=1$

# Model Calibration

The model is calibrated using an approximate Bayesian computation sequential Monte Carlo (ABC SMC) algorithm^1^ to give 1,000 parameter sets. At the first iteration of the ABC SMC, parameters were sampled from their prior distributions (Table 1 in main paper). At subsequent iterations, the parameter sets from the previous iteration were sampled from with weights dependent upon the prior likelihood of the parameter set and the perturbation kernel (uniform in this implementation). The sampled parameter sets were perturbed using a uniform perturbation kernel, which could perturbate each parameter by at most +/- 5% of the prior range, so as to still be within the prior ranges, accepting those that gave model fits whose goodness of fit, measured by the log-likelihood, was less than that iteration’s tolerance. At each iteration of the ABC SMC, the tolerance was set to be the 75th percentile of the goodness of fits of accepted model fits in the previous iteration. The adaptive tolerances guaranteed a monotonically decreasing sequence of tolerances. The ABC SMC routine was stopped when the tolerances began to stabilise (<0.5% relative difference between successive iterations). The 1,000 parameter sets obtained at the end of the routine were then used directly to parameterise the transmission dynamics of the full model. Model fits and data used to calibrated the model are shown in supplementary figures 1-6.


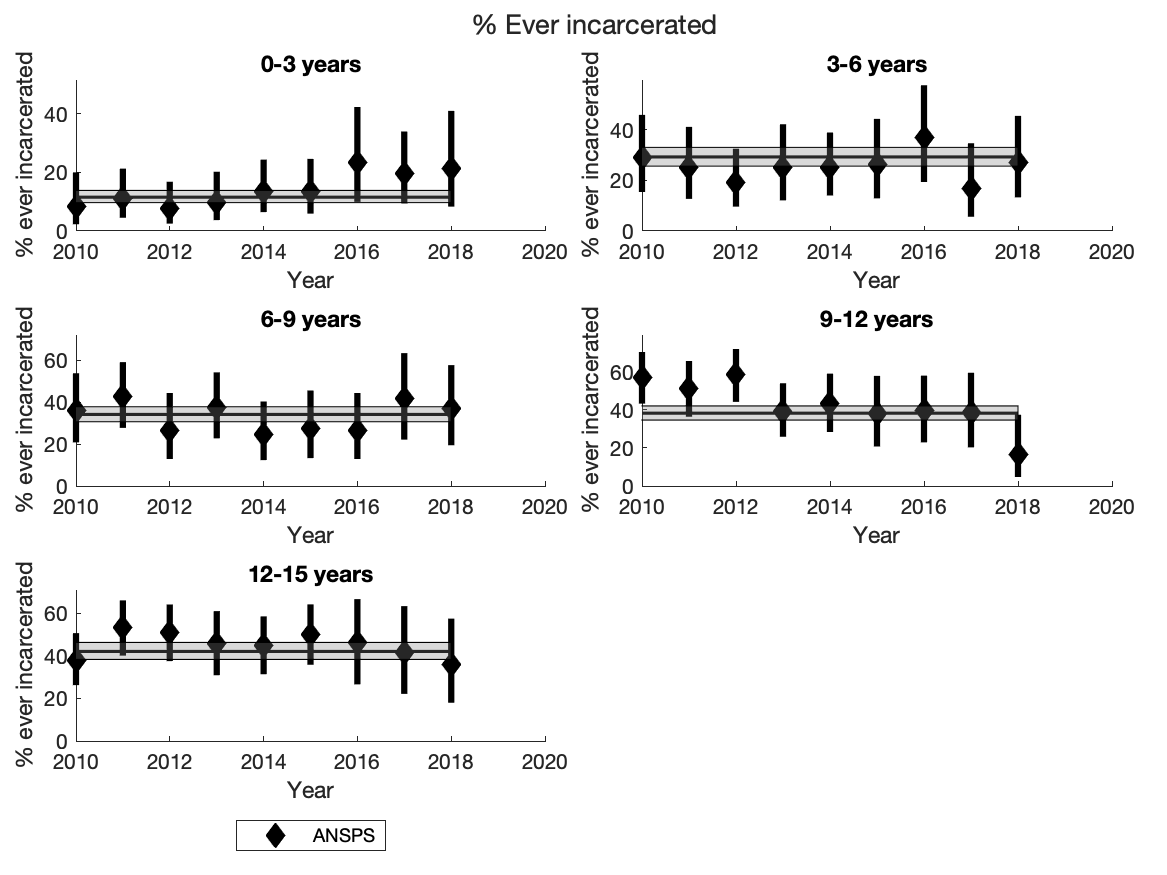


**Supplementary Figure 1:** Comparison of model fits and calibration data for the proportion of

community PWID who have ever been incarcerated by duration of injecting. Lines show the median model projections whilst the shaded area shows the 95%CrI for the baseline projections. Data points from ANSPS used in model calibration with their 95%CI are shown for comparison.


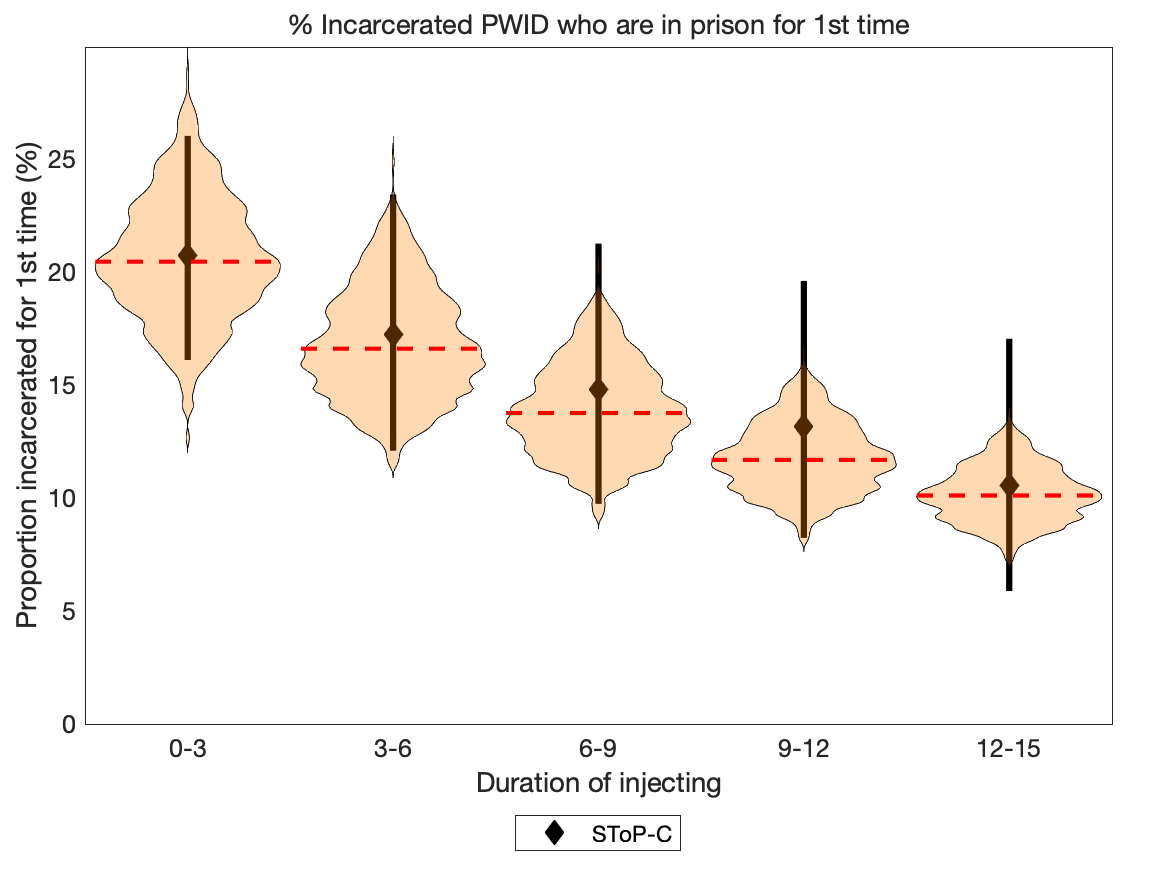


**Supplementary Figure 2:** Comparison of model fits and calibration data for the proportion of

proportion of incarcerated PWID who are incarcerated for first time by duration of injecting. Dashed red Lines show the median model projections whilst the shaded area shows the distribution of model projections. Data points from SToP-C used in model calibration with their 95%CI are shown for comparison.


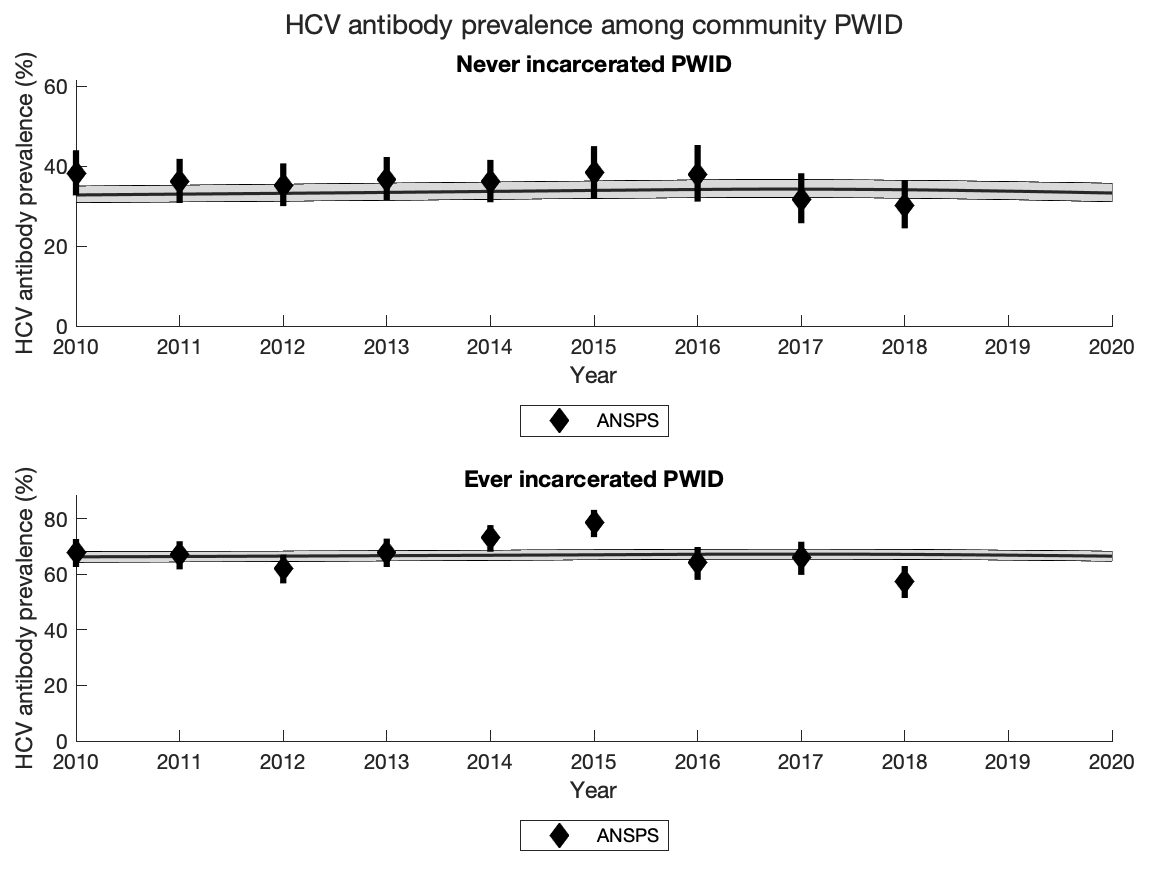


**Supplementary Figure 3:** Comparison of model fits and calibration data for HCV antibody prevalence proportion among community PWID by incarceration history (never incarcerated and ever incarcerated). Lines show the median model projections whilst the shaded area shows the 95%CrI for the baseline projections. Data points from ANSPS used in model calibration with their 95%CI are shown for comparison.


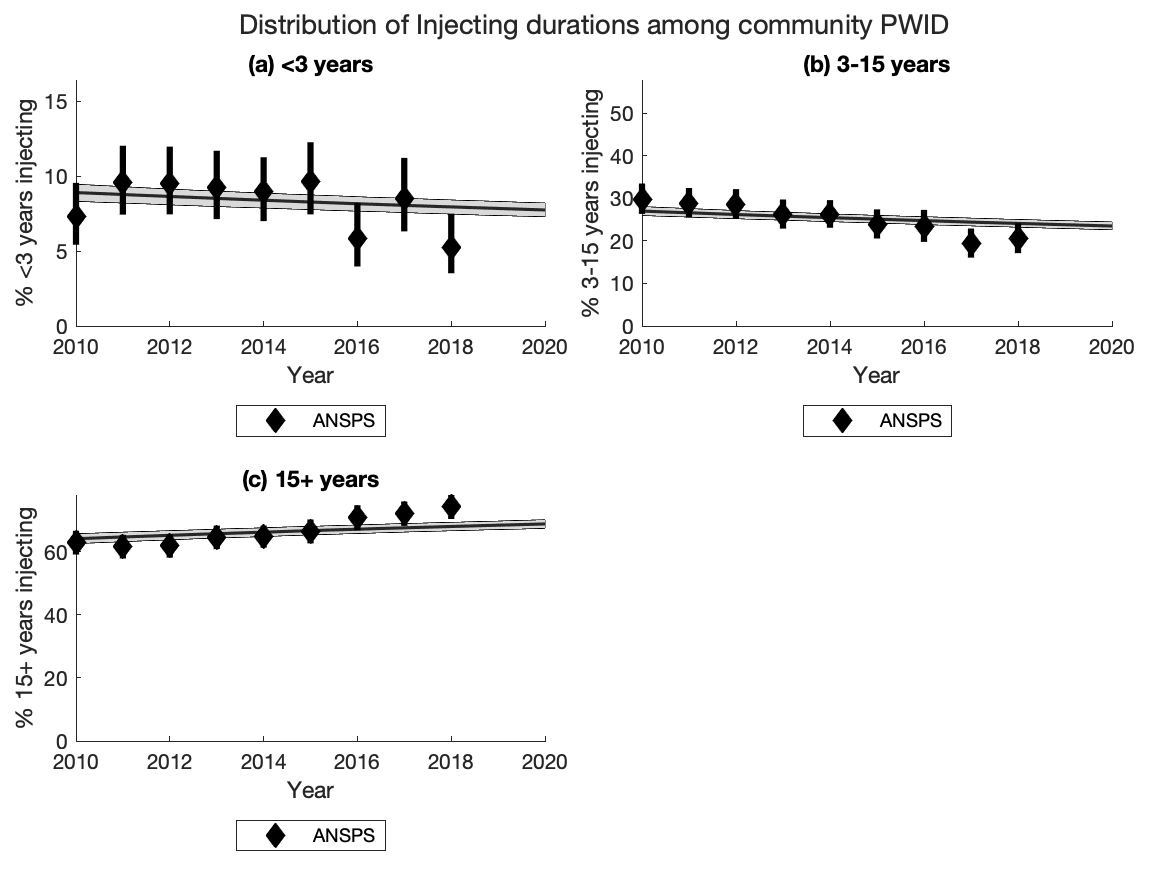


**Supplementary Figure 4:** Comparison of model fits and calibration data for the proportion of PWID in each duration of injecting category. Lines show the median model projections whilst the shaded area shows the 95%CrI for the baseline projections. Data points from ANSPS used in model calibration with their 95%CI are shown for comparison.


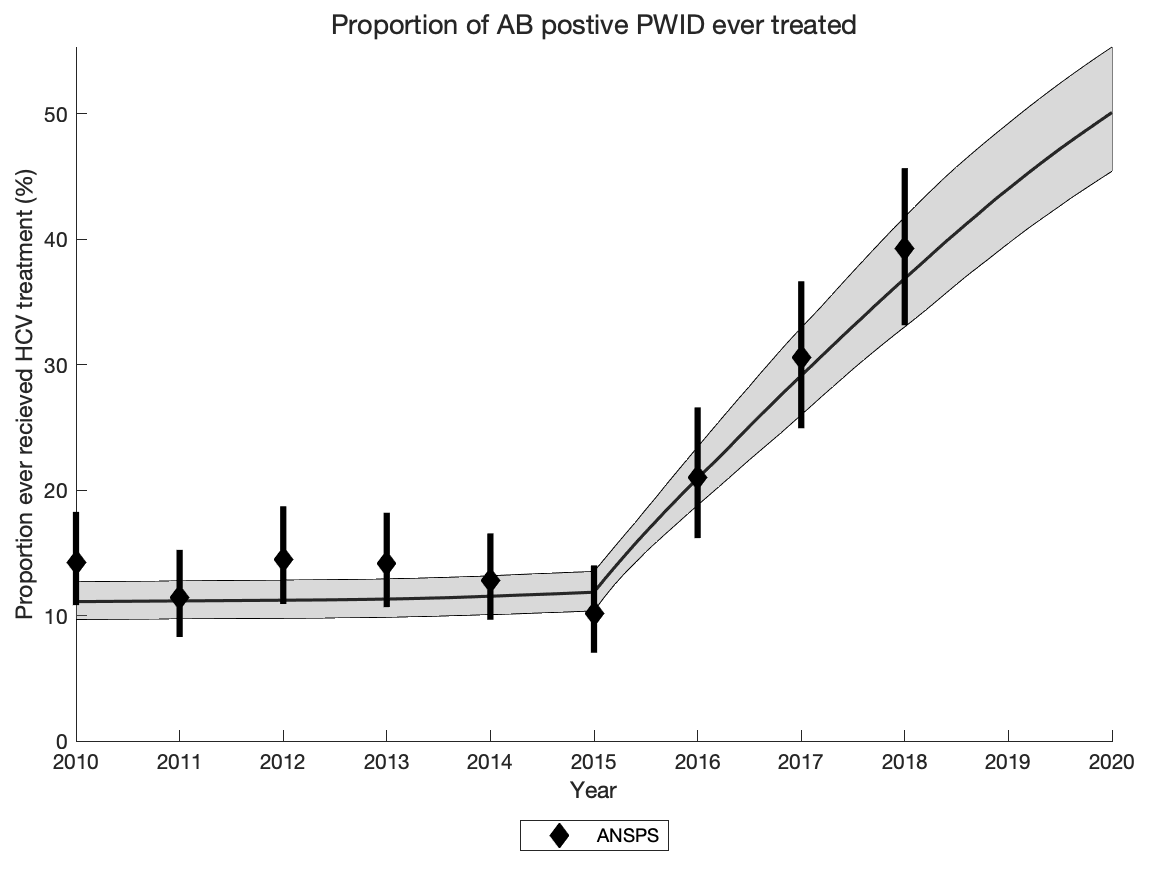


**Supplementary Figure 5:** Comparison of model fits and calibration data for the proportion of HCV antibody positive PWID who have ever been treated. Lines show the median model projections whilst the shaded area shows the 95%CrI for the baseline projections. Data points from ANSPS used in model calibration with their 95%CI are shown for comparison.


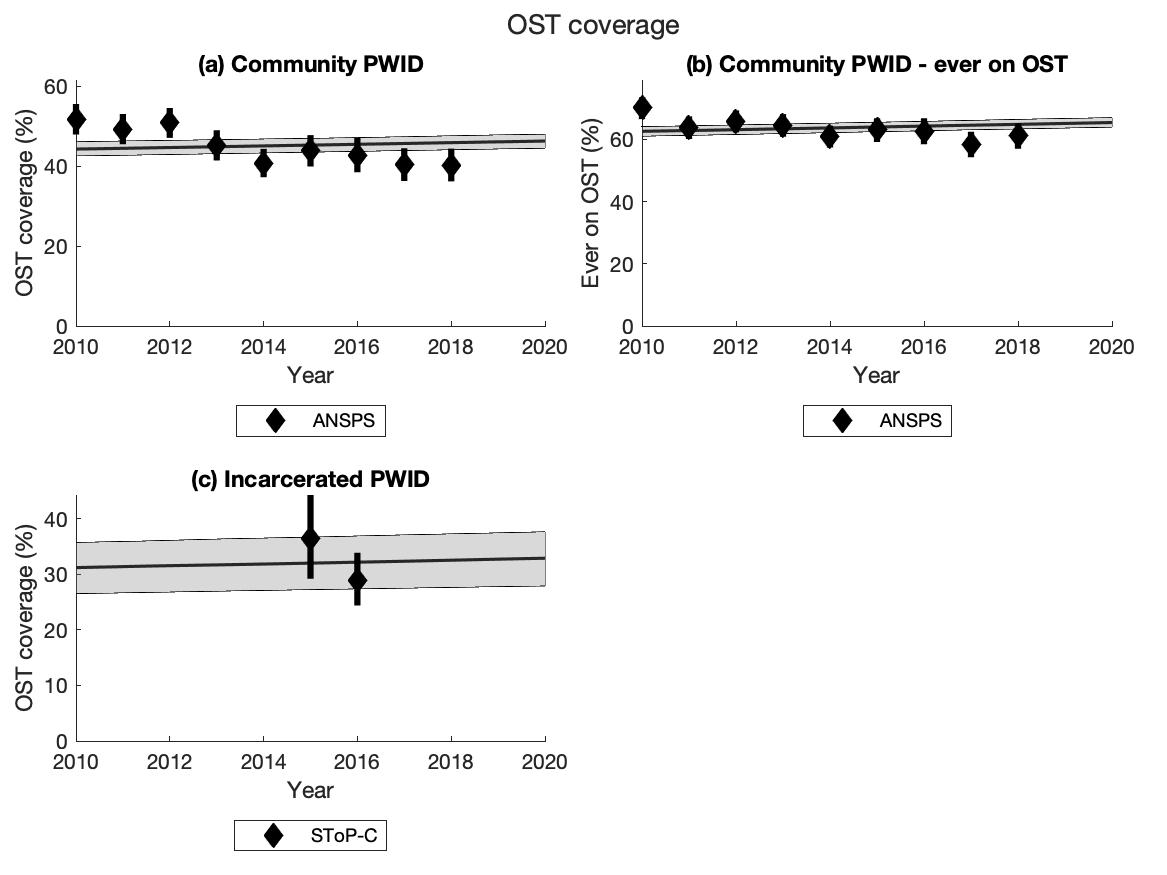


**Supplementary Figure 6:** Comparison of model fits and calibration data for OAT coverage. Lines show the median model projections whilst the shaded area shows the 95%CrI for the baseline projections. Data points from ANSPS (community) and SToP-C (prison) used in model calibration with their 95%CI are shown for comparison.

# Modelling the Introduction of Prison-Based NSP

The model is used to analyse the impact of introducing prison-based NSP from 2020 onwards at 50% or 100% coverage. We assume that NSP reduces HCV transmission risk (‘the effectiveness of NSP’) by 56% (95%CI 20-76%)[19]). This is modelled by reducing the force of infection in prison ($\beta_{p})$ by a factor that considers the coverage ($c$, as a proportion) and effectiveness ($e$, as a proportion) of NSP. The modified force of infection, $\beta_{p}^{NSP}$ is given by:

$${\beta_{p}^{NSP}=\beta}_{p}(1-c+c(1-e))$$

# Analysis of Covariance

Supplementary Table 1: Contribution of parameters to uncertainty in model projections of the impact of prison-based interventions to reducing HCV incidence over 2020-2030. Only parameters contributing >1% of uncertainty are shown.

| **Parameter** | **Contribution to the total uncertainty** |
| --- | --- |
| Community HCV treatment rate from 2015 onwards | **56.2%** |
| Relative increase in rate of initiating HCV treatment if on OAT | **21.9%** |
| Annual re-incarceration rate among PWID >=3 years injecting | **7.8%** |
| Prison HCV treatment rate from 2020 onwards | **5.0%** |
| Annual incarceration rate among PWID >=3 years injecting | **3.3%** |
| Relative increase in HCV transmission risk if currently incarcerated PWID | **1.6%** |
| Relative increase in HCV transmission risk amongst recently released PWID (12 months since release) | **1.1%** |

**References**

1. Toni T, Welch D, Strelkowa N, Ipsen A, Stumpf MP. Approximate Bayesian computation scheme for parameter inference and model selection in dynamical systems. *J R Soc Interface* 2009; **6**(31): 187-202.
